# Non-communicable diseases: mapping research funding organisations, funding mechanisms and research practices in Italy and Germany

**DOI:** 10.1186/s12961-017-0249-x

**Published:** 2017-10-02

**Authors:** Victor Stephani, Silvia Sommariva, Anne Spranger, Oriana Ciani

**Affiliations:** 1 Department of Health Care Management, Technical University Berlin, Berlin, Germany; 2 CERGAS - Università Commerciale L. Bocconi, Milan, Italy; 3 Evidence Synthesis and Modelling for Health Improvement, University of Exeter Medical School, Exeter, United Kingdom

**Keywords:** Non-communicable diseases, Biomedical research, Germany, Italy, Cross-country comparison, Funding

## Abstract

**Background:**

Evidence shows that territorial borders continue to have an impact on research collaboration in Europe. Knowledge of national research structural contexts is therefore crucial to the promotion of Europe-wide policies for research funding. Nevertheless, studies assessing and comparing research systems remain scarce. This paper aims to further the knowledge on national research landscapes in Europe, focusing on non-communicable disease (NCD) research in Italy and Germany.

**Methods:**

To capture the architecture of country-specific research funding systems, a three-fold strategy was adopted. First, a literature review was conducted to determine a list of key public, voluntary/private non-profit and commercial research funding organisations (RFOs). Second, an electronic survey was administered qualifying RFOs. Finally, survey results were integrated with semi-structured interviews with key opinion leaders in NCD research. Three major dimensions of interest were investigated – funding mechanisms, funding patterns and expectations regarding outputs.

**Results:**

The number of RFOs in Italy is four times larger than that in Germany and the Italian research system has more project funding instruments than the German system. Regarding the funding patterns towards NCD areas, in both countries, respiratory disease research resulted as the lowest funded, whereas cancer research was the target of most funding streams. The most reported expected outputs of funded research activity were scholarly publication of articles and reports.

**Conclusions:**

This cross-country comparison on the Italian and German research funding structures revealed substantial differences between the two systems. The current system is prone to duplicated research efforts, popular funding for some diseases and intransparency of research results. Future research will require addressing the need for better coordination of research funding efforts, even more so if European research efforts are to play a greater role.

**Electronic supplementary material:**

The online version of this article (doi:10.1186/s12961-017-0249-x) contains supplementary material, which is available to authorized users.

## Background

Research investments in Europe have shown an increase in the past years, with the expenditure for research and development, as a percentage of gross domestic product, having grown from 1.8% to 2.0% between 2002 and 2014, although still falling short of the 3% target set under the Europe 2020 strategy [1]. Biomedical, and more specifically, non-communicable disease (NCD) research funding show cross-country differences in structures and funding amounts, and there is still a long way to go towards an integration of research efforts across the European Union [2]. In fact, studies show that territorial borders, both political and language-related, continue to have an impact on research collaboration in Europe [3]. Knowledge of national research structural contexts is therefore crucial to the promotion of Europe-wide policies for research funding. Nevertheless, characteristics of national research systems that drive funding decision and research outcomes are not well understood and the expertise to navigate through national research landscapes scarcely travels from one country to another. This paper aims to deepen the knowledge on national funding systems for biomedical research, with a focus on NCDs in Europe, taking into consideration two national case studies, Italy and Germany. Both are major European Union continental countries that nevertheless differentiate in terms of their healthcare system and biomedical research.

Germany operates a Statutory Health Insurance system, which is mainly funded through contributions from employers and employees. In Italy, the National Health Service provides universal coverage and is funded through general taxation and, in smaller part, through user charges. In terms of biomedical research systems, both countries have a track record of excellence and internationally recognised work, albeit with some differences in the approach to research funding (e.g. higher level of private and industry-funded research activities in Germany). Therefore, the Italian and German research systems appear to be a good match to understand the multilevel framework of research funding, its funding patterns, and its mechanisms and actors.

## Methods

### Definitions

This article builds on the findings of the project MAPPING_NCD (grant agreement #602536), a 2-year project funded by the European Commission under the 7th framework programme, that aimed to draw a map of European research funding for five NCD areas in order to develop evidence-based policies for future research. NCDs are defined as diseases that are not passed from person to person and are of chronic nature [4]. The NCD areas considered herein are cardiovascular diseases, chronic respiratory diseases, mental health diseases, diabetes and cancer. A previous publication provides details on the mixed methods approach, combining qualitative and quantitative data, employed throughout the project [5]. An inclusive definition of ‘research’ that encompasses basic, clinical, policy and management research was adopted in this analysis.

### Study design

This mixed-method study uses a case-oriented approach [6] to describe the NCD research funding systems in Italy and Germany. The comparative research design is a two-country comparison, which is often associated with mixed methodologies [7], as employed in the analysis of the two country-specific systems. Similarly to a study conducted by Viergever and Hendriks [8] on public and philanthropic institutions funding health research globally, three dimensions were considered, namely identification of the network of research funding organisations (RFOs), assessment of funding patterns and funding distribution mechanisms in Italy and Germany.

### Data collection methods

To capture the architecture of country-specific research funding systems with a broad-based view, three steps were used. First, a non-systematic literature review was conducted to determine a list of key public, voluntary/private non-profit and commercial RFOs at national level and their funding practices in biomedical research, with focus on NCD-related projects. PubMed and Google scholar were searched for relevant articles based on disease-specific keywords (e.g. ‘diabetes’, ‘funding’ combined with ‘Italy’). Second, an electronic survey was administered to qualify funding by individual RFOs, defined as those funding NCD-related research activities worth at least €0.5 million during the period 2002–2013, as determined by analysis of public financial statements released by the organisations. This threshold was set in order to select organisations playing a relatively significant role in shaping the research stream within particular disease areas. The survey was designed using Qualtrics® (Provo, UT) and translated in the national language to encourage RFO participation [5]. Contact with RFOs was initiated in early April 2014 via email and was followed-up several times by phone. Third, survey results were integrated with semi-structured interviews with key opinion leaders (KOLs) purposively selected to reflect a range of factors, including expertise in conducting research, geographic location or expertise in awarding research funding. Transcripts were analysed qualitatively using inductive thematic analysis [9]. The guide investigated the themes of research activities and funding, research priorities, duplication of research efforts and role of the European Union. Additionally, expectations regarding outputs of funded research projects were investigated with surveyed RFOs and KOLs. A constant comparative approach was used to iteratively identify emerging themes that contribute to generate a vision about future research funding strategies to tackle gaps and challenges currently perceived by experts in the field [10].

## Results

### The Italian case

#### Network of research funding organisations

**Fig. 1:**
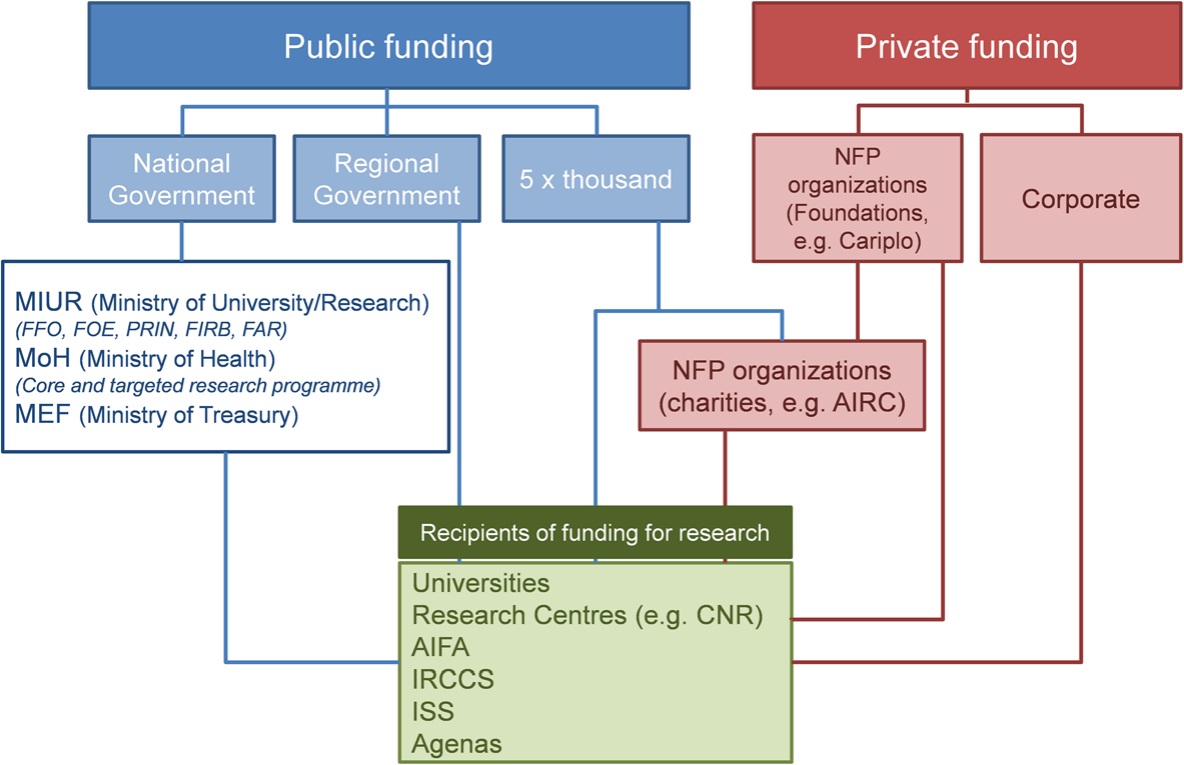
Funding for biomedical research in Italy. *FFO* Fondo di Finanziamento Ordinario (Fund for Ordinary Financing), *FOE* Fondo Ordinario per il Finanziamento degli Enti e Istituzioni di Ricerca (Ordinary Fund for Financing of Research Institutes), *PRIN* Progetti di Ricerca di Interesse Nazionale (Research Projects of National Interest), *FIRB* Fondo per gli Investimenti della Ricerca di Base (Fund for Investments in Basic Research), *FAR* Fondo per le Agevolazioni alla Ricerca (Fund for Incentives for Research), *NFP* not-for-profit, *IRCCS* Istituto di Ricovero e Cura a Carattere Scientifico (Scientific Institute for Research, Hospitalisation and Healthcare), *ISS* Istituto Superiore di Sanità (National Institute for Health), *AIFA* Agenzia Italiana del Farmaco (National Drug Agency)

The documentary analysis revealed a fragmented network of biomedical research funding where central public bodies operate alongside regional authorities and the private sector (Fig. 1). Public sector funding is organised via three main channels, namely the national government, the regional governments and the ‘5 per thousand’ mechanism, which allows tax payers to donate part of their taxable income to a selected charitable organisation [11]. Private sources include corporate funding, as well as non-profit organisations, either charities or foundations tied to the banking sector.

Forty-six RFOs met the funding level criteria (Additional file 1: Table S1), of which 5 (10.9%) were national institutions (such as the Department of Health), 21 (45.6%) were regional institutions representative of as many Regions and Autonomous Provinces in Italy, and 11 (23.9%) were banking foundations. The remaining 9 (19.6%) were research associations (e.g. Italian Association for Cancer Research) or scientific societies (e.g. Italian Society of Cardiology). Of the invited RFOs, data were collected for 26 organisations (57%), of which 14 were private non-profit organisations and 12 were public RFOs. Three follow-up interviews with KOLs were conducted with the aim to solicit views and experiences of people involved in both the conduct and funding of research in Italy. All KOLs were experienced Principal Investigators working in hospital settings.

#### Mechanisms used to distribute funding and expectations regarding outputs

The analysis of the funding allocation process revealed that 44% of the RFOs surveyed indicated government or other public funding as their major source of income, with a relatively minor role of fundraising and private donations. Banking foundations reported financial income as their primary source. Thematic analysis of KOL interviews highlighted a related challenge with regards to the financial problems posed by decreasing available public funding for biomedical research. Informants also emphasised the competition among sponsors and research teams to get access to adequate level of funding, which tends to put team leaders under pressure to secure continuous funding, fostering challenges in human resources management practices. Most RFOs reported the funding process to be unstructured and accept unsolicited proposals from researchers. In the view of one KOL, these calls were often “*open, to reward quality rather than steer research*” (Interview #1). Only two RFOs (8%) reported establishing priorities through a process of consultation with other stakeholders. Additionally, there seemed to be a tendency to value continuity with past funding decisions.

Coherently with the funding mechanisms highlighted, most RFOs reported not having pre-defined expectations for grant receivers. The most reported expected output was the publication of academic articles. However, informants categorised this particular output as a propaedeutic step, with the definition of impact going beyond academic publishing.

Most of the public resources were dedicated to Italian or Italian-based researchers and research organisations since the aim was to strengthen national scientific basis in order to promote effective collaboration in large international and European research initiatives.

#### Funding patterns towards biomedical and NCD research

**Fig. 2:**
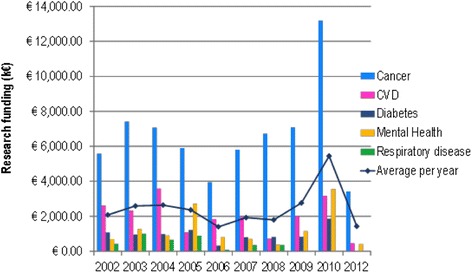
Research projects of national interest (PRIN) funding by non-communicable disease area 2002–2012 [23]. No funding data available for 2011

In Italy, public funding is distributed from the central government, from Regions or through the ‘5 per thousand’ mechanism. The Ministry for Education, University and Research organises funding through different channels, namely the Fondo di Finanziamento Ordinario (General University Fund), which consisted of €6.7 billion in 2013, mainly for permanent staff salaries and with a minor proportion allocated for research; the Fondo Ordinario per il Finanziamento degli Enti e Istituzioni di Ricerca (Research Centres Fund), addressed to research centres managed by the Ministry for Education, University and Research itself (147 in total, of which 10 are health related), with a funding of €1.7 billion for 2013; the Fondo per gli Investimenti della Ricerca di Base (Supplementary Fund for Basic Research) to finance universities and research centres working in cooperation with industry and amounting to less than €100 million; the Fondo per le Agevolazioni alla Ricerca (Fund for Incentives for Research) for corporate research (less than €100 million); and Progetti di Rilevante Interesse Nazionale (research projects of national interest). Data for the latter funding channel are project specific, they were analysed and categorised according to the NCDs of interest (Fig. 2).

**Fig. 3:**
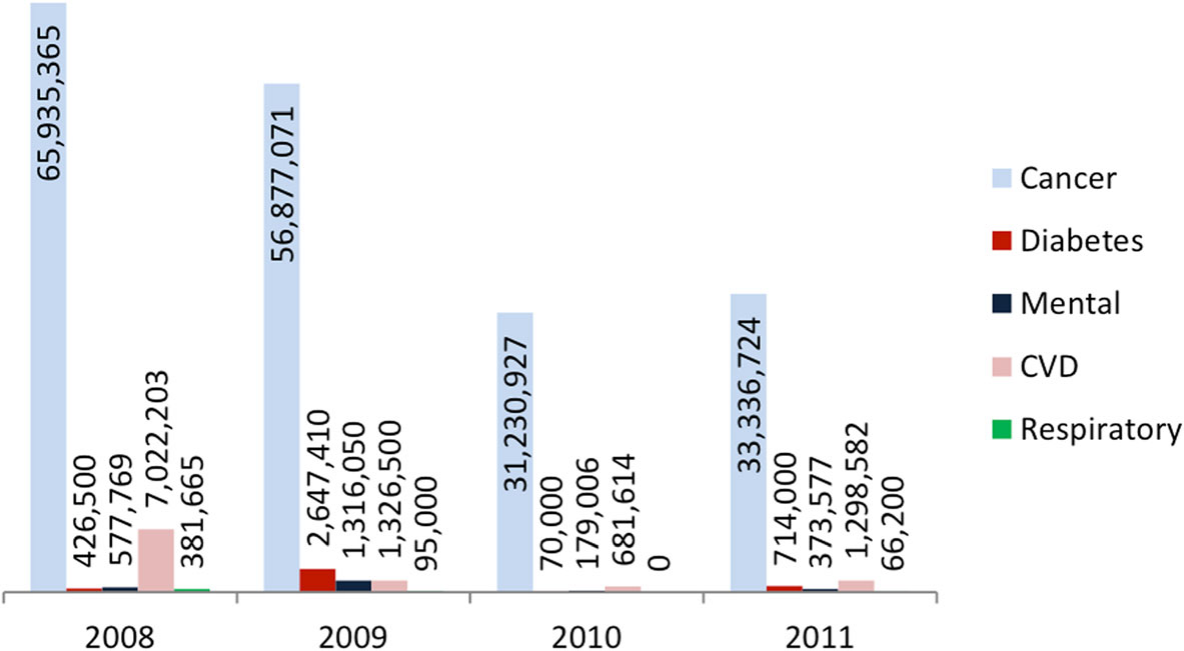
Non-communicable disease funding via ‘5 per thousand’ channel, 2008–2011 [24]

Regional funding per NCD could not be mapped due to lack of data, as also noted by Compagni and Tediosi [12]. As regards the ‘5 per thousand’ donations, data are available for the period 2008–2011. A proportion of approximately 15% of all entities (i.e. 40 to 45 organisations from 2008 to 2011) receiving funds has not made a report available. The remaining contributions have been allocated to different NCD areas according to the project titles (Fig. 3).

**Fig. 4:**
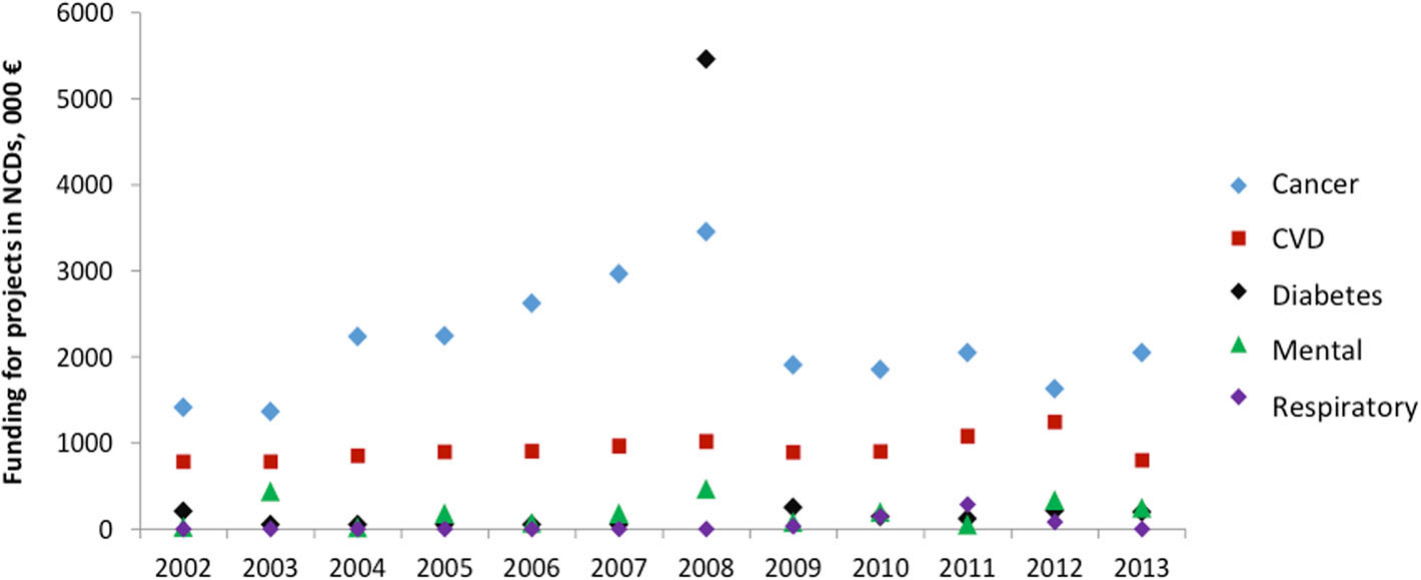
Projects financed by banking foundations in non-communicable diseases, total amount 2002–2013 (survey data). Thirteen research funding organisations reported data for 2003, 2004, 2006, 2011, 2012, 2013; 14 for 2009; 16 for 2007 and 2008; 15 for 2005; and 10 for 2002

According to the Italian Association of Bank Foundations and Mutual Savings, bank foundations financed scientific and technological research with €128.3 million, equal to 14% of the total activities funded in 2013, and financing over 1200 projects. Approximately 30% (€38.9 million, corresponding to 344 projects) were allocated to healthcare research, which represents an area of increasing investments (+60% compared to the previous year). Figure 4 shows the amount of funding by banking foundations as retrieved through the survey.

### The German case

#### Network of research funding organisations

Public research funding in Germany is organised among the federal government and 16 federal states (*Länder*). Länder are responsible for providing higher research institutions with institutional/core funding, which is not tied to any prerequisite or priority, but is instead determined as global budgets. However, in addition to core funding, (public) project funding has become an important source of funding for universities and other research performing bodies over the last years [13].

The key organisation for project-based funding is the German Research Foundation (DFG), which is financed by the federal government and Länder alike (58:42 share). The DFG does not have a particular research focus and provides funds to all fields of science and humanities [14].

Alongside the DFG, ministries on federal government (and Länder) level are the second pillar in terms of public project funding. The main ministry for federal project funding is the ministry for education and research (BMBF) and accounts for almost 60% of all ministry funds. Biomedical, and therefore NCD, research is also funded by the Ministry of Health in certain areas of ministerial interests, but this accounts for only 4% of all research grants [13]. All ministries authorise external institutions for distributing, coordinating and evaluating their project funds. For health-related project funds, the German Aerospace Centre is responsible for administering research grants.

Private non-profit and commercial RFOs include corporate funding as well as foundations. There are several corporate RFOs of relevant size, tied to companies such as Volkswagen or Robert Bosch, but also hundreds of smaller non-profit foundations. However, the foundation landscape in Germany is very fragmented, organised around specific diseases or acknowledged gaps in medical care or to help the family of a specific group of patients. Its share on the overall funding on biomedical research is not well documented, but is expected to rank third next to public and commercial funding [15]. The majority of identified foundations were not eligible for inclusion because of their limited investments in NCD research.

Within this framework, 13 RFOs were identified as eligible for inclusion in our analysis (Additional file 2: Table S2). Of these, 10 are private foundations, in most cases linked to bigger companies. The remaining three are public national institutions. Of the invited RFOs, full data was collected for 30% (three public (DFG, German Aerospace Centre, which represents BMBF, and Bundesministerium für Gesundheit (Federal Ministry of Health)) and one private). Seven follow-up interviews with KOLs were conducted with the aim to solicit views and experiences of people involved in both the conduct and funding of research in Germany.

#### Mechanisms used to distribute funding and expectations regarding outputs

The major source of income for public RFOs are the government and other public sources. Private foundations receive their funding from company revenues and/or through charity. Fundraising plays a minor role for big foundations – smaller foundations rely on private donations. Interviews with KOLs revealed the importance of small foundations for bridging financial gaps (e.g. between two bigger publicly funded projects). In general, KOLs involved in conducting research mentioned the increasing competition among research grants and the time burden for researchers to write proposals and to acknowledge varying deadlines, grant proposal guidelines and other prerequisites. Some KOLs highlighted the positive effect of competition in research, provided that there is transparency and only excellence is rewarded.

All surveyed RFOs reported establishing priorities through a process of consultation with other stakeholders. For example, the DFG claims having over 15,000 scientific reviewers per year for consultation [14]. Coherently with the funding mechanisms reported, most RFOs reported having expectations, such as published articles or reports, from grant receivers. The DFG, as the key public funding organisation, reported having much greater expectations, such as improvements in (drug) therapies, increased political attention for a particular research field or even legislative changes.

Both the DFG and the ministries distribute the majority of their projects to national research organisations, but are expanding their involvement in international research funding and fellowships for young researchers going abroad (e.g. cooperation with developing countries funded by DFG).

#### Public funding patterns towards biomedical and NCD research

**Fig. 5:**
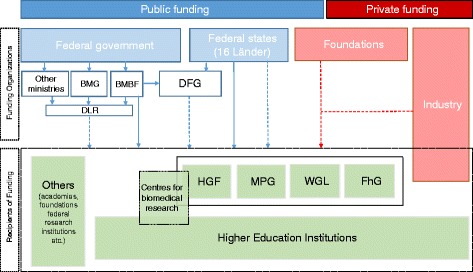
Non-communicable disease research landscape in Germany. Dashed arrows indicate project funding flows, continuous lines symbolise core funding. *DFG* Deutsche Forschungsgemeinschaft (German Research Foundation), *BMG* Bundesministerium für Gesundheit (Federal Ministry of Health), *BMBF* Bundesministerium für Bildung und Forschung (Federal Ministry of Education and Research), *DLR* Deutsches Zentrum für Luft- und Raumfahrt (German Aerospace Center), *HGF* Helmholtz-Gemeinschaft Deutscher Forschungszentren (The Helmholtz Association of German Research Centres), *MPG* Max Plank Gesellschaft (Max Planck Society), *WGL* Leibniz Gemeinschaft (Leibniz Association), *FhG* Fraunhofer Gesellschaft (Fraunhofer Society)

**Fig. 6:**
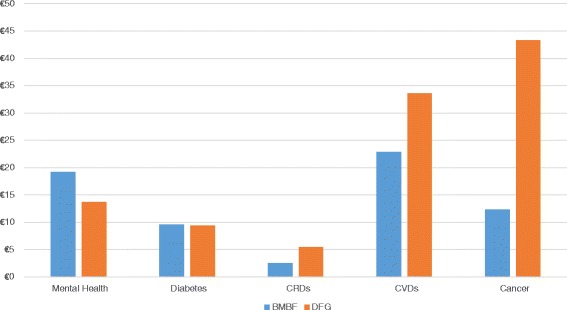
Public project funding (million Euro) on non-communicable disease research in Germany in 2012

In 2013, the BMBF and DFG distributed €66.5 million (out of a total budget of €3.3 bn) and €105.2 million (total budget of €2.7 bn), respectively, towards NCD projects (not only directed to universities, but also to other research performing actors; Fig. 5) [13, 14]. Figure 6 depicts how the NCD money was shared among the five major disease areas. The DFG had a particular focus on cancer research, while the BMBF put a higher emphasis on cardiovascular disease (CVD) research. Chronic respiratory disease research received the lowest share of total funding. However, all clinicians interviewed expressed worry about the continuous trend of popular crowding for some NCDs without surveying research grants against the actual disease burden or foreseeable future trends (e.g. CVD research suffers from an underfunding of research compared to cancer, although CVDs cause the highest disease burden). Additionally, the fragmentation of available funding options poses an enormous administrative burden to gain an overview of possible funding options and its required formalities.

KOLs stated that the focus has been on basic research grants in the last years and emphasised the missing link to applied research in the NCD field. They highlighted the severity of translational gaps in NCD research. In the process from ‘bench to bedside’, there are several well-known barriers and disincentives for not completing the full research cycle in Germany (Interview #3 and #7). Therefore, the government started a new initiative, the so called health research centres, which receive continuous funding [16]. The research centres (five centres for each NCD area plus one centre for infectious disease) put an emphasis on translational science and are meant to bridge several phases of NCD research while providing a longer funding perspective. Some interviewed partners valued these newly established networks across several layers of the fragmented German research landscape (e.g. between public universities and Helmholtz Association of Research centres, which is the largest scientific organisation in Germany) and stated that they offer a platform for consolidated cooperation along highly specialised research areas (Interview #3 and #7). On the other hand, the considerable funding for these research centres is shared amongst only some of the research organisations and its outcome is yet to be seen (Interview #5 and #6).

## Discussion

A varying degree of fragmentation was observed across the two analysed research systems; the number of included RFOs in Italy is almost four times greater than that in Germany (Table 1). The German funding system appears to be more concentrated than the Italian system, where the network of actors, both public and private, is more comprehensive, mostly due to the numerous banking foundations. In Germany, public project funding is mainly distributed through the DFG and the ministries, and in particular the BMBF, both of which act on a national level. Regional funding bodies are not as important as in Italy. A comparable mechanism to the ‘5 per thousand’ mechanism does not exist in Germany. Private persons have to actively donate part of their income; although private donations lead to tax breaks, only 6% of German citizens donate into health research, compared to 39% in Italy (European Union average is 26%) [17]. Our analysis shows that a considerable share of money for NCD research has been donated by Italian citizens over the last years. However, one issue of this funding mechanism is the unpredictability of future funding budgets (Fig. 3).

**Table 1:** Cross-country comparison of non-communicable disease funding systems in Italy and Germany

	Italy	Germany
Identified eligible RFOs	46	13
Main research funding sources	Central and regional governments, ‘5 per thousand’ mechanism, foundations (banking)	Central government and other national funding bodies, foundations (charity, company)
Public NCD project funding/total project funding budget	PRIN: €4.1 mn/€100 mn (2012)	DFG: €105.2 bn/€2.7 bn (2013)
	‘5 per thousand’: €35mn (2011)	BMBF: €66.5 bn/€3.3 bn (2013)
Public NCD funding	Cancer (€37.1 mn)	Cardiovascular diseases (€56.4 mn)
	Cardiovascular diseases (€1.4 mn)	Cancer (€55.6 mn)
	Diabetes (€710 k)	Mental health (€32.8 mn)
	Mental health (€550 k)	Diabetes (€18.9 mn)
	Chronic respiratory diseases (€66 k)	Chronic respiratory diseases (€7.9 mn)

*BMBF* Bundesministerium für Bildung und Forschung (Federal Ministry of Education and Research), *DFG* Deutsche Forschungsgemeinschaft (German Research Foundation), *NCD* non-communicable disease, *PRIN* Progetti di Rilevante Interesse Nazionale (research projects of national interest), *RFO* research funding organisation

It appears that the majority of funding grants in Italy and Germany is distributed without explicit research priorities. In Italy, only 8% of analysed RFOs make research investments through a process of consultation. In Germany, the largest analysed research foundation – the German DFG – stated that there is no explicit process of deciding research priorities for most of their funding. Therefore, future funding decisions should also reflect actual societal needs (e.g. higher investments into CVD research, since they account for 42% of all deaths in Europe [18]). Moreover, the fragmented system and the missing consultation process is prone for research duplication.

The international collaboration in research is still underrepresented in both countries. Most funding agencies distribute their money to national research agencies, but are expanding their involvement in international research funding (e.g. as part of the so called High-Tech strategy in Germany) [19]. Smaller countries in Europe tend to have a higher share of international research collaboration than larger countries such as Italy and Germany [20].

KOLs from both countries shed light on the final perception of the users of funds, highlighting the increasing competition among researchers to secure continuous funding. Furthermore, high bureaucratic barriers and the average time it takes for research funding organisations to decide over applications were identified as major challenges for researchers. In Germany, the importance of small foundations for bridging these financial gaps were highlighted. Another major concern is the gap between basic and clinical research. New initiatives, such as the establishment of the German health research centres (one research centre per NCD) or the National Plan for Chronic Illnesses in Italy (i.e. a plan for harmonising all preventive and therapeutic interventions for patients with chronic illnesses), are attempting to overcome this particular gap, but their outcome is yet to be seen.

Due to a lack of data and difficulties to receive information of several RFOs, many NCD research funding investments could not be mapped, especially from the private sector (e.g. private foundations in Germany). In Italy, however, private banking funding appears to be much lower than money gained through public channels such as the ‘5 per thousand mechanism’. In Germany, total private third party funding has decreased over the last decade and it is estimated that it accounts for less than 10% of the total research funding at Higher Education Institutions [15]. Public funding appears to be the major source for research in both countries.

### Limitations

This study provides an attempt to comprehensively map the funding landscape for NCD research in two large European countries. Despite the mixed methodologies employed with regards to communication with RFOs during the project, we relied on publicly available data or on the willingness to cooperate by the RFOs. However, because of lack of reporting and data in this field, particularly in the private sector, it is possible that some of the RFOs active in either country were not identified and were therefore not included in the analysis. We selected a specific framework to perform the cross-country evaluation, taking into account the profound structural differences between the two systems. Other approaches could have been used in the comparison, such as an indirect way of estimating research funding based on the analysis of research outputs (i.e. published academic papers) [21]; however, this effort was not immediate to apply and operationalise across five disease areas. This analysis does not include any measures of research outcome as bibliometric data, and therefore no statements on the quality or impact of research can been made on either German and Italian research.

## Conclusion

This analysis of Italian and German research funding structures revealed substantial differences between the two systems, making any crude statements about NCD research in the two countries biased if not adjusted for appropriate factors.

If the European Union wants to proceed with its European Research Area, as declared in the Lisbon Treaty [22], more cross-countries information about current research system differences and their characteristics is needed. Even with more funding available for research efforts, we need to obtain a greater understanding of how and by what principles the funds are administered by national RFOs. The particularities of the national research landscapes in Italy and Germany have been constant over the years, providing several advantages and disadvantages. More coordination of research priorities between European Union member states on NCDs and their funding institutions is required. The current system is prone to duplicated research efforts, fund crowding for certain diseases and member states, and the intransparency of research results. This requires greater attention and understanding of research system differences by politicians, RFOs, researchers and other stakeholders.

## Additional files

Additional file 1: Table S1. List of included Italian RFOs. (DOCX 16 kb)

Additional file 2: Table S2. List of included German RFOs. (DOCX 15 kb)

## Abbreviations

BMBF: Bundesministerium für Bildung und Forschung (Federal Ministry of Education and Research)
CVD: Cardiovascular disease
DFG: Deutsche Forschungsgemeinschaft (German Research Foundation)
DLR: Deutsches Zentrum für Luft- und Raumfahrt (German Aerospace Center)
FAR: Fondo per le Agevolazioni alla Ricerca (Research Support Fund)
KOL: Key opinion leader
NCDs: non-communicable diseases
RFO: Research funding organisation
