# Supplementary material for: Non-communicable diseases: mapping research funding organisations, funding mechanisms and research practices in Italy and Germany
Source: Health Res Policy Syst. 2017 Oct 2;15:85. doi: 10.1186/s12961-017-0249-x (PMC5625614; doi:10.1186/s12961-017-0249-x)
Supplement: Supplementary file 1 — List of included Italian RFOs. (DOCX 16 kb) [file 12961_2017_249_MOESM1_ESM.docx]

Additional file 1: Table S1: List of excluded Italian RFOs

| **#** | **Name** |
| --- | --- |
| 1 | Italian Ministry of Health |
| 2 | Italian Drug Agency |
| 3 | Nationl Council for Research |
| 4 | National Institute for Health |
| 5 | Ministry of Research, Education and University |
| 6 | Abruzzo Region |
| 7 | Basilicata Region |
| 8 | Calabria Region |
| 9 | Campania Region |
| 10 | Emilia Romagna Region |
| 11 | Friuli Venezia Giulia Region |
| 12 | Lazio Region |
| 13 | Liguria Region |
| 14 | Lombardia Region |
| 15 | Marche Region |
| 16 | Molise Region |
| 17 | Piemonte Region |
| 18 | Puglia Region |
| 19 | Sardegna Region |
| 20 | Sicilia Region |
| 21 | Toscana Region |
| 22 | PA Bolzano Autonomous Province |
| 23 | PA Trento Autonomous Province |
| 24 | Umbria Region |
| 25 | Valle d'Aosta Region |
| 26 | Veneto Region |
| 27 | Fondazione Banco di Sardegna (Banking foundation) |
| 28 | Fondazione Cariparma (Banking foundation) |
| 29 | Fondazione Cassa di Risparmio di Cuneo (Banking foundation) |
| 30 | Fondazione Cassa di Risparmio di Genova e Imperia (Banking foundation) |
| 31 | Fondazione Cassa di Risparmio di Lucca (Banking foundation) |
| 32 | Fondazione Cassa di Risparmio di Perugia (Banking foundation) |
| 33 | Fondazione Cassa di Risparmio di Puglia (Banking foundation) |
| 34 | Fondazione Cassa di Risparmio di Verona Vicenza Belluno e Ancona (Banking foundation) |
| 35 | Fondazione del Monte di Bologna e Ravenna (Banking foundation) |
| 36 | Fondazione Monte dei Paschi di Siena (Banking foundation) |
| 37 | Fondazione Roma (Banking foundation) |
| 38 | AIRC Italian Association for Cancer Research |
| 39 | Telethon |
| 40 | LILT Italian League for the Fight against Cancer |
| 41 | FISM Italian Foundation on Multiple Sclerosis |
| 42 | Fondazione Piemontese per la Ricerca sul Cancro (Piemontese Foundation for Cancer Research) |
| 43 | Istituto Nazionale per la Ricerca sul Cancro (National Institute for Cancer Research) |
| 44 | Fondazione Umberto Veronesi |
| 45 | Fondazione Italiana di Ricerca sulla SLA (Foundation for Research on ALS) |
| 46 | Associazione Italiana per la Ricerca sul Diabete (Italian Association for Research on Diabetes) |
