# Supplementary material for: Non-communicable diseases: mapping research funding organisations, funding mechanisms and research practices in Italy and Germany
Source: Health Res Policy Syst. 2017 Oct 2;15:85. doi: 10.1186/s12961-017-0249-x (PMC5625614; doi:10.1186/s12961-017-0249-x)
Supplement: Supplementary file 2 — List of included German RFOs. (DOCX 15 kb) [file 12961_2017_249_MOESM2_ESM.docx]

Additional file 2: Table S2: List of included German RFOs

| **#** | **Name** |
| --- | --- |
| 1 | Volkswagen (VW) Stiftung |
| 2 | Robert Bosch Stiftung GmbH |
| 3 | Bertelsmann Stiftung |
| 4 | Baden-Wurttemberg Stiftung GmbH |
| 5 | Die Dietmar Hopp Stiftung |
| 6 | GHST: Gemeinnützige Hertie-Stiftung zur Förderung von Wissenschaft, Erziehung, Volks- und Berufsbildung |
| 7 | Die Klaus Tschira Stiftung |
| 8 | Fritz Thyssen Stiftung |
| 9 | Dr. Werner Jackstädt Foundation |
| 10 | Wilhelm Sander Stiftung |
| 11 | Deutsche Forschungsgemeinschaft |
| 12 | BMBF - Bundesministerium für Bildung und Forschung |
| 13 | BMG - Bundesministerium für Gesundheit |
